# Assessing the individual risk of fecal poliovirus shedding among vaccinated and non-vaccinated subjects following national health weeks in Mexico

**DOI:** 10.1371/journal.pone.0185594

**Published:** 2017-10-12

**Authors:** Leticia Ferreyra-Reyes, Luis Pablo Cruz-Hervert, Stephanie B. Troy, ChunHong Huang, Clea Sarnquist, Guadalupe Delgado-Sánchez, Sergio Canizales-Quintero, Marisa Holubar, Elizabeth Ferreira-Guerrero, Rogelio Montero-Campos, Mauricio Rodríguez-Álvarez, Norma Mongua-Rodriguez, Yvonne Maldonado, Lourdes García-García

**Affiliations:** 1 Instituto Nacional de Salud Pública, Cuernavaca, Morelos, México; 2 Eastern Virginia Medical School, Norfolk, Virginia, United States of America; 3 Stanford University School of Medicine, Stanford, California, United States of America; 4 Facultad de Medicina, Universidad Nacional Autónoma de México, Ciudad de México, México; Instituto de Salud Carlos III, SPAIN

## Abstract

**Background:**

Mexico introduced inactivated polio vaccine (IPV) into its routine immunization (RI) schedule in 2007 but continued to give trivalent oral polio vaccine (tOPV) twice a year during national health weeks (NHW) through 2015.

**Objectives:**

To evaluate individual variables associated with poliovirus (PV) shedding among children with IPV-induced immunity after vaccination with tOPV and their household contacts.

**Materials and methods:**

We recruited 72 children (both genders, ≤30 months, vaccinated with at least two doses of IPV) and 144 household contacts (both genders, 2 per household, children and adults) between 08/2010 and 09/2010 in Orizaba, Veracruz. Three NHW took place (one before and two after enrollment). We collected fecal samples monthly for 12 months, and tested 2500 samples for polioviruses types 1, 2 and 3 with three serotype-specific singleplex real-time RT-PCR (rRT-PCR) assays. In order to increase the specificity for OPV virus, all positive and 112 negative samples were also processed with a two-step, OPV serotype-specific multiplex rRT-PCR.

**Analysis:**

We estimated adjusted hazard ratios (HR) and 95% CI using Cox proportional hazards regression for recurrent events models accounting for individual clustering to assess the association of individual variables with the shedding of any poliovirus for all participants and stratifying according to whether the participant had received tOPV in the month of sample collection.

**Results:**

216 participants were included. Of the 2500 collected samples, using the singleplex rRT-PCR assay, PV was detected in 5.7% (n = 142); PV1 in 1.2% (n = 29), PV2 in 4.1% (n = 103), and PV3 in 1.9% (n = 48). Of the 256 samples processed by multiplex rRT-PCR, PV was detected in 106 (PV1 in 16.41% (n = 42), PV2 in 21.09% (n = 54), and PV3 in 23.05% (n = 59). Both using singleplex and multiplex assays, shedding of OPV among non-vaccinated children and subjects older than 5 years of age living in the same household was associated with shedding of PV2 by a household contact. All models were adjusted by sex, age, IPV vaccination and OPV shedding by the same individual during the previous month of sample collection.

**Conclusion:**

Our results provide important evidence regarding the circulation of poliovirus in a mixed vaccination context (IPV+OPV) which mimics the “transitional phase” that occurs when countries use both vaccines simultaneously. Shedding of OPV2 by household contacts was most likely the source of infection of non-vaccinated children and subjects older than 5 years of age living in the same household.

## Introduction

The generalized and sustained use of the two polio vaccines available to prevent paralytic poliomyelitis, the inactivated poliomyelitis vaccine (IPV) and the oral poliomyelitis vaccine (OPV), has significantly decreased the incidence of this illness since the World Health Assembly declared its intent to eliminate poliomyelitis in 1988. The main risk for polio today is concentrated in the two countries that are still endemic (Afghanistan and Pakistan) and in some countries where there may be instability in maintaining national health systems (Nigeria and Laos) [1]. In industrialized countries where it has been possible to control the disease, the risk is less; however, poliomyelitis will not be globally eradicated as long as the virus still circulates in any part of the world [2–4]. In the year 2013 the Global Poliomyelitis Eradication Initiative (GPEI) published the “Strategic plan for the eradication of polio: 2013–2018” to consolidate the containment and eradication of all wild type polioviruses (WTP), attenuated viruses contained in OPV (Sabin and OPV-like) and those developed from continued circulation of attenuated poliovirus from the OPV vaccine (VDPV) [5].

In Mexico, IPV is used as part of a pentavalent vaccine that also includes diphtheria and tetanus toxoids and acellular pertussis adsorbed, since August 2007. Four doses are given to all infants as part of the universal vaccination scheme at 2, 4, 6 and 18 months of age. Additionally, all children older than 6 months and 5 years of age and younger that have received at least two doses of IPV received one dose of trivalent OPV (tOPV) through 2016 in the National Health Weeks (NHW) that are carried out in February and May every year; bOPV replaced tOPV in 2017.

The main risk factor for the development of vaccine derived poliomyelitis virus (VDPV) is the continued routine use of OPV. The elimination of VDPVs as well as other OPV-like viruses depends on cessation of use of OPV. Because wild type poliovirus 2 (WTP2) was last known to be transmitted in 1999 and was declared eradicated in December 2015, and the only cases due to poliovirus type 2 have been caused by VDPV2, the international community agreed to remove the Sabin 2 virus from tOPV starting in May 2016. Only bivalent OPV is used (with Sabin strains 1 and 3) (bOPV) in a combined scheme with IPV in all the countries that previously used OPV [5].

The success of this plan relies on understanding the factors associated with duration and patterns of OPV-derived virus circulation in a community vaccinated with IPV. We have previously published results on the duration and pattern of OPV-derived virus circulation [6, 7]. The present study had the purpose of further identifying the individual risk factors for OPV-derived virus shedding.

## Materials and methods

### Study population, enrolment and follow up

We carried out a prospective, observational cohort study, recruiting 72 children and 144 household contacts in four communities in Orizaba, Veracruz, Mexico. The details of the methodology (inclusion and exclusion criteria, sample collection and immunization history) have been described previously [6]. Briefly, enrollment of participants took place from 25 August to 22 September, 2010 and participants were followed for 12 months. Three NHW took place around these dates: 29 May to 4 June 2010, 15 to 19 February, 2011 and 28 May to 3 June 2011.

### Stool sample analysis

Collection, transport and processing of samples have been previously described [6]. Briefly, stools samples were kept in a cooler until brought to the local laboratory, separated into cryovials and stored at −80°C. Samples were processed in laboratories at the Stanford University School of Medicine (US), in Stanford, CA, and at East Virginia Medical School (EVMS) in Norfolk, VA. Both laboratories have the necessary conditions to handle these types of samples according to the third Global Action Plan to minimize the risk associated with laboratories (GAP III) [8]. At Stanford and EVMS stool underwent RNA extraction, reverse transcription and real-time polymerase chain reaction (rRT-PCR) to look for vaccine poliovirus serotypes 1, 2 and 3 according to previously published methods [9].

In order to increase the specificity for OPV virus, 256 samples, including all 144 samples that were positive for at least one poliovirus serotype and 112 randomly selected negative samples using the above described rRT-PCR assays were processed using a two-step, serotype-specific multiplex rRT-PCR that was adapted from the literature [10, 11] and described in [12].

### Statistical analysis

The primary outcome was fecal poliovirus shedding in collected samples. We considered participants to be OPV vaccinated when they had received OPV vaccine in the NHW previous to date of sample collection. According to Mexican guidelines, children were eligible for OPV if they were 5 years old or younger and had received at least two doses of IPV. Household contacts could be any age and have any vaccination history but had to reside in the same household as the enrolled child. We used the terms “non-vaccinated” or “not vaccinated” to include non-vaccinated 5 years old or younger children that had not been vaccinated during NHW previous to sample collection and subjects older than 5 years of age living in the same household. We compared the characteristics of tOPV vaccinated individuals in any of the 3 NHW with individuals who were not vaccinated using the Chi-square or Kruskal–Wallis tests using p<0.05 at two tails for statistical significance. Percent positive stool samples containing each serotype from tOPV vaccinated and non-vaccinated persons were compared using the binomial test. We used Kaplan-Meier curves adjusting by participant to assess positive sample probability for each OPV serotype according to whether the person did or did not receive OPV in the past NHW and used the log-rank test to detect significant differences (p<0.05).

We compared samples with and without OPV strains according to whether at least one household contact of any age shed poliovirus during the same month of sample collection using X^2^ test. We conducted this comparison in all samples and stratified according to whether the studied sample belonged to an individual who had been tOPV vaccinated in the last NHW or had not. The association of individual characteristics with OPV shedding (any type) during follow-up period was analyzed by using a Cox proportional hazards regression for recurrent events model accounting for individual clustering. In this model, the different time periods to each event for the same subject are analyzed separately and adjusted for the fact that time periods within each subject are independent. Since results of the gamma shared frailty model to consider within-individual random effect were not statistically significant, we did not include them [13]. We conducted these analyses for all samples, for those from children 36 months old and younger and for the subgroup of 256 samples that were processed with multiplex rRT-PCR. For the model for children 36 months old and younger, we substituted having received OPV vaccine during NHW as a categorical variable (yes/no) for number of OPV doses received during lifetime. All variables were entered into the Cox proportional hazards regression models according to statistical significance (p<0.2) or biological plausibility, and non-significant variables were removed sequentially. Statistical analyses were performed using the software Stata 13.1.

### Ethical considerations

The study protocol was approved by the Ethics, Biosafety, and Research Committees of the Instituto Nacional de Salud Publica (FWA00015605), by the Public Health Center of Orizaba, Veracruz, Mexico, the Stanford University Institutional Review Board, and the Eastern Virginia Medical School (EVMS) Institutional Review Boards. Written informed consent was obtained from participants, parents or guardians.

## Results

We enrolled 216 participants (110 children younger than 5 years of age (31 pairs in the same household) and 106 household contacts older than 5 years of age in the 72 households. Characteristics of participants according to OPV vaccination during the three NHW around the study period are shown in Table 1. Thirty-seven percent of the study population was male, median age was 9.6 (Interquartile range (IQR) 2.2–26.6) years of age; 50% lived in rural areas. Environmental sanitation was limited as indicated by 52.8% and 11.1% of participants living in households without indoor plumbing and with dirt floor, respectively. Fourteen percent (31/216) of participants were underweight. Among children under 2 years of age, 31.6% (36/114) were breastfeeding. Median number of IPV doses received previous to enrollment or during the study among all participants was 3 (Interquartile range (IQR) 0–0). As expected, vaccinated individuals were younger and more likely to shed OPV during study period. They were also more likely to breastfeed, to have received IPV previous to enrollment or during the study period and to have lower body mass index. There were no differences between vaccinated and non-vaccinated participants regarding rural versus urban households or households without indoor plumbing or dirt floor.

**Table 1:** Characteristics of study participants (Orizaba, Veracruz, Mexico, May 2010 to August 2011).

Characteristic	Total	Vaccinated with OPV in prior NHW	Not vaccinated	p-value^a^
	No. (%)	No. (%)	No. (%)	
Shedding any serotype	23/216 (10.6)	19/45 (42.2)	4/171 (2.3)	<0.001
Male	80/216 (37.0)	22/45 (48.9)	58/171 (33.9)	0.064
Age (years) (Median, IQR)	9.6 (2.2–26.6)	1.8 (1.5–2.4)	19.9 (7.1–28.8)	<0.001^b^
*Age group*				
<3 years-old	80/216 (37.0)	45/45 (100.0)	35/171 (20.5)	<0.001
3 to 5 years-old	30/216 (13.9)	0/45 (0.0)	30/171 (17.5)	
>5 years old	106/216 (49.1)	0/45 (0.0)	106/171 (62.0)	
Rural (*vs* urban) residence	108/216 (50.0)	25/45 (55.6)	83/171 (48.5)	0.402
Residence without indoor plumbing	114/216 (52.8)	24/45 (53.3)	90/171 (52.6)	0.933
Residence with dirt floor	24/216 (11.1)	5/45 (11.1)	19/171 (11.1)	1.000
Body mass index (Mean) (SD)	20.8 (5.64)	17.8 (4.24)	21.8 (20.9–22.7)	<0.001^c^
*Body mass index category*				
Underweight	31/216 (14.4)	2/45 (4.4)	29/171 (17.0)	0.002
Normal weight	85/216 (39.4)	12/45 (26.7)	73/171 (42.7)	
Overweight and obesity	100/216 (46.3)	31/45 (68.9)	69/171 (40.4)	
Breastfeeding (only for children under 2 years of age)	36/114 (31.6)	23/43 (53.5)	13/71 (18.3)	<0.001
Number of IPV doses received previously or during the study (median) (IQR)	3 (0–0)	3 (3–4)	0 (0–0)	<0.001^b^

^a^ χ2 test;

^b^ Kruskal-Wallis;

^c^ t- Student test;

NA, Not applicable; IQR, interquartile range.

**Fig 1:**
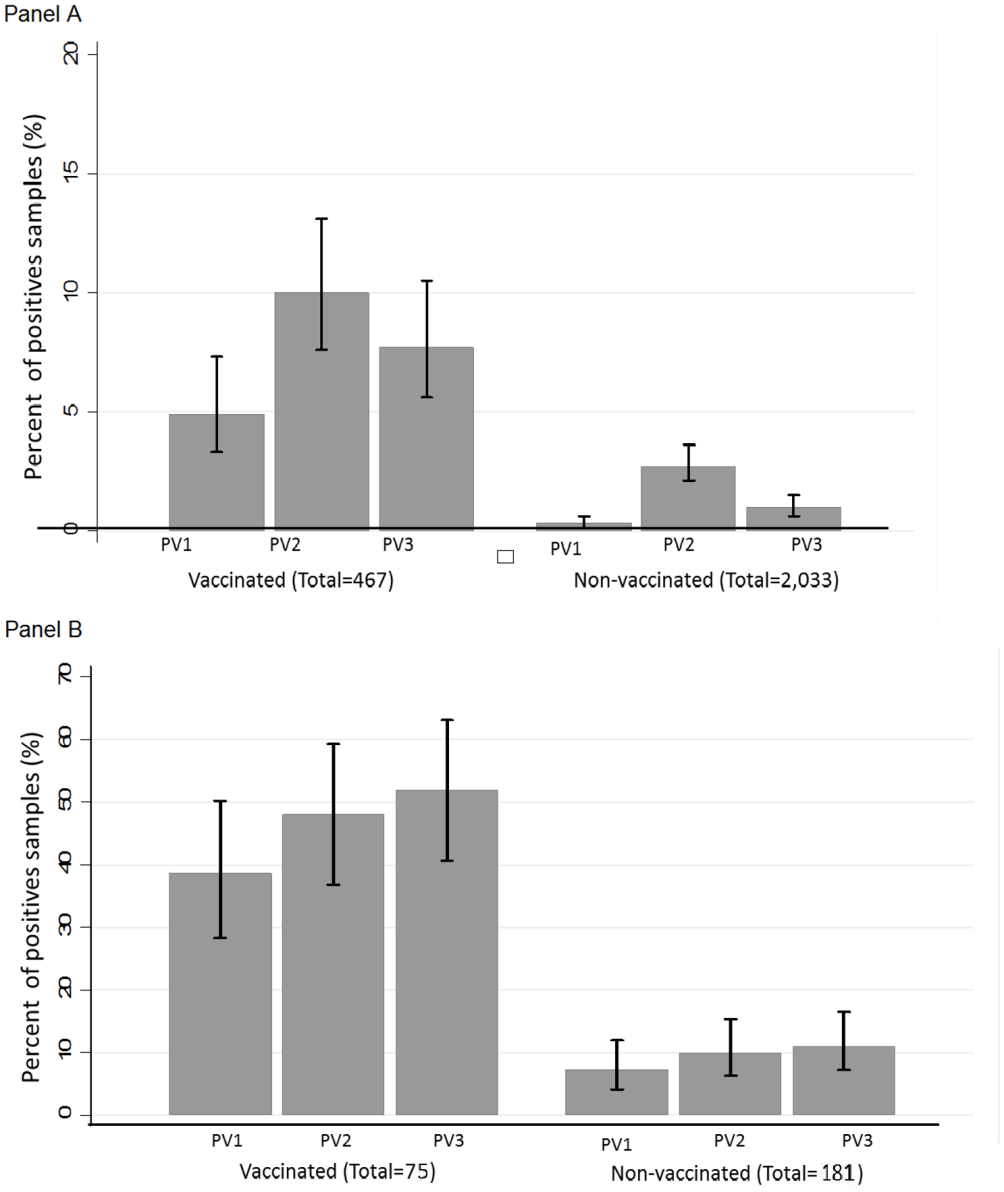
Percentage of positive stool samples with each serotype according to whether the person received tOPV in the NHW previous to sample collection. **Samples processed by singleplex (Panels A) and multiplex rRT-PCR (Panel B) assays**. Panel A. Among stools from vaccinated individuals, serotype 2 was more frequent than serotype 1(10.1% versus 4.9%, p = 0.003). No differences were observed between serotype 1 compared to 3 or 2 compared to 3. In contrast, among stools samples from persons who did not receive tOPV in the past NHW, Sabin serotype 2 was more frequent than Sabin 1 (2.8% versus 0.3%, p<0.001) and Sabin 3 (2.8% versus 0.6%, p<0.001). There were no differences between serotype 1 versus 3 (0.3% versus 0.6%, p = 0.156). Panel B. We did not find differences for frequency of OPVs in samples from vaccinated or non-vaccinated individuals.

During the study period we collected 2500 samples from the 216 participants. Of the 2500 samples; using singleplex rRT-PCR we detected OPV strains in 142 (5.7%) (Sabin 1 = 1.2% [29/2500]; Sabin 2 = 4.1% [103/2500] and Sabin 3 = 1.9% [48/2500]. Among the 467 samples obtained from participants who had been vaccinated with OPV in the previous NHW, we detected poliovirus Sabin 1 in 23 samples (4.9%); Sabin 2 in 47 samples (10.1%) and Sabin 3 in 36 samples (7.7%). In the 2033 samples obtained from participants who were not vaccinated with OPV in the previous NHW, we detected Sabin poliovirus 1 in 6 samples (0.3%); Sabin 2 in 56 samples (2.8%) and Sabin 3 in 12 samples (0.6%). Among stool samples from individuals who received tOPV in the previous NHW, Sabin poliovirus type 2 was more frequent than serotype 1 (10.1% versus 4.9%, p = 0.003). There were no differences between serotype 1 versus 3 (4.9% versus 7.7%, p = 0.08) and serotype 2 versus 3 (10.1% versus 7.7%, p = 0.25). In contrast, among stools samples from persons who did not receive tOPV in the past NHW, Sabin serotype 2 was more frequent than Sabin 1 (2.8% versus 0.3%, p<0.001) and Sabin 3 (2.8% versus 0.6%, p<0.001). There were no differences between serotype 1 versus 3 (0.3% versus 0.6%, p = 0.156). On the 256 samples processed by the multiplex rRT-PCR, we detected OPV strains in 106 (41.4%) (Sabin 1 = 16.41%% [42/256]; Sabin 2 = 21.09% [54/256] and Sabin 3 = 23.05% [59/256]. In the 75 samples obtained from participants who had been vaccinated with OPV in the previous NHW, we detected poliovirus Sabin 1 in 29 samples (38.7%); Sabin 2 in 36 samples (48.0%) and Sabin 3 in 39 samples (52.0%). In the 181 samples obtained from participants who were not vaccinated with OPV in the previous NHW, we detected Sabin poliovirus 1 in 13 samples (7.2%); Sabin 2 in 18 samples (9.9%) and Sabin 3 in 20 samples (11.1%). There were no differences according to type of OPV either in vaccinated or non-vaccinated individuals. Percent positive stool samples containing each serotype according to whether the person received tOPV in the previous NHW and rRT-PCR are shown in Fig 1.

**Fig 2:**
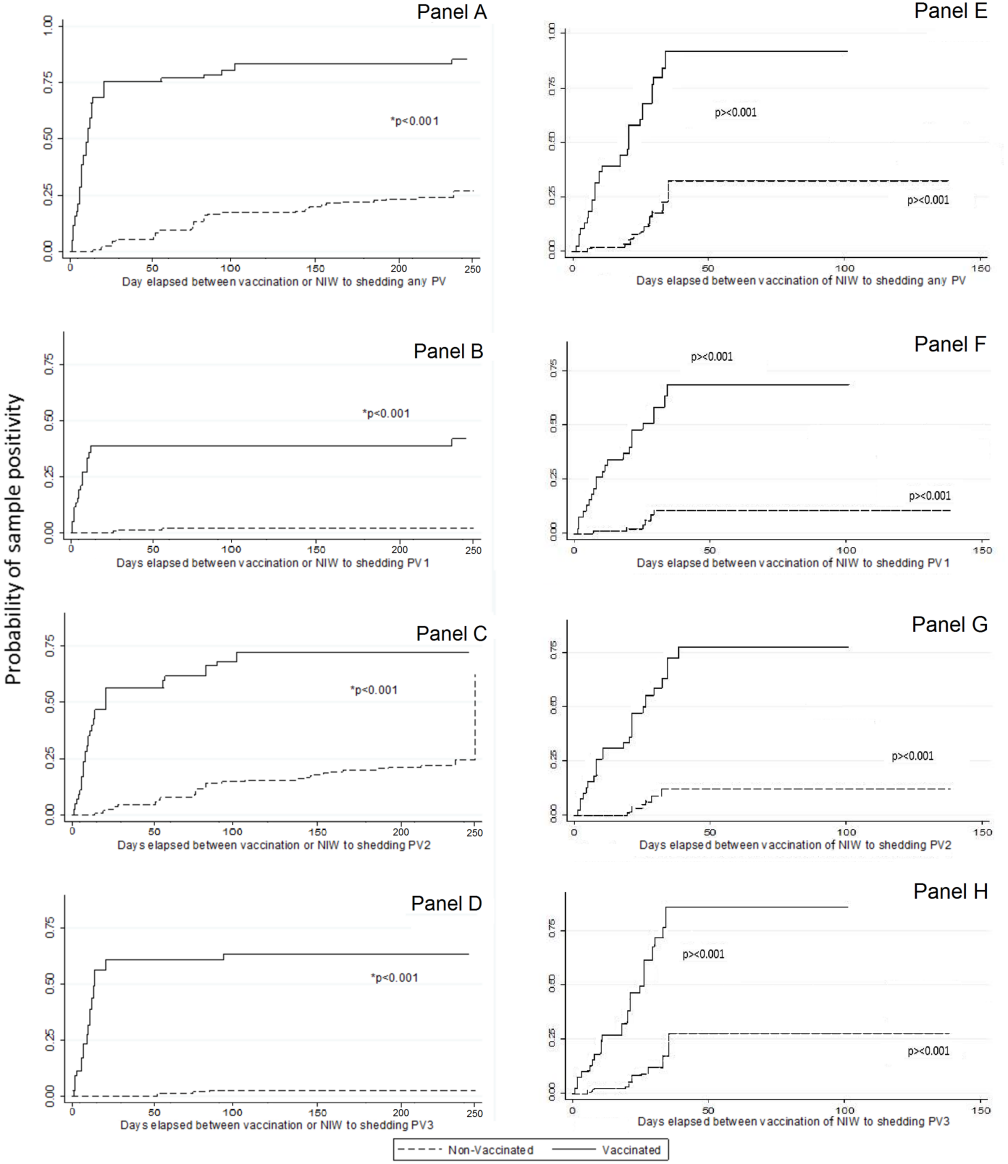
Probability of positive stool samples according to each serotype of OPV and stratified according to vaccination in the previous NHW before the sample collection. Singleplex assay: Panel A. Any Poliovirus; Panel B, OPV1; Panel C, OPV2, Panel D, OPV3. Multiplex assay; Panel E. Any Poliovirus; Panel F, OPV1; Panel G, OPV2, Panel H, OPV3 The probability of sample positivity was higher among samples from tOPV vaccinated individuals as compared to samples from non-vaccinated individuals for all serotypes and using both assays. (p<0.001, Kaplan Meier test adjusted for each individual). Using singleplex assay, comparison of positive probability rates revealed that curves for each serotype were not significantly different among vaccinated individuals (panels B, C, D solid lines) whereas Sabin 2 curve was significantly different from Sabin 1 and 3 curves among non-vaccinated individuals (panels B, C, D dotted lines). Vaccinated individuals had higher positivity rate during the first 30 days for all serotypes, whereas duration of OPV2 shedding was longer among non-vaccinated individuals. Using multiplex assay, comparison of positive probability rates revealed that curves for each serotype were not significantly different among vaccinated individuals (panels E, F, G, H solid lines). Among non-vaccinated individuals, positivity rates slightly higher and longer for Sabin 3 (panels E, F, G, H dotted lines).

As expected, probability of identification of poliovirus was higher in samples obtained from vaccinated individuals than in samples from non-vaccinated persons for all serotypes and using both assays (p<0.001, Log-rank test adjusted by individual), Fig 2. When samples were processed by singleplex assay, comparison of positive probability rates among samples from vaccinated individuals revealed that curves for serotypes 1 and 3 reached a plateau around day 30 (Fig 2, panels B and D, solid lines) while rates for serotype 2 (Fig 2, panel C, solid line) reached a plateau at around day 100. Among samples from non-vaccinated individuals, Sabin 2 curve was also significantly different from Sabin 1 and 3 curves; its rate of increase was very much like the rate of increase in the unvaccinated group (Fig 2, panels B, C, D dotted lines). The sudden late rise in the unvaccinated type 2 group was due to shedding of one of two individuals who were followed up to 240 days. Their samples were collected on January 9th, 2011 (before the NHW which took place from February 15 to 19, 2011). When samples were processed by multiplex assay, highest probability of positive rates was observed for Sabin 3. Among vaccinated individuals plateau was reached at approximately the same interval, while for non-vaccinated individuals the latest time to the plateau was reached by Sabin 3 curve. We did not observe extended shedding for any OPV among samples processed by multiplex assay.

Table 2 shows bivariate association between identification of each serotype of OPV in at least one sample from a household contact during the same month of sample collection and shedding by all participants, vaccinated children and non-vaccinated individuals testing with singleplex (Panel A) and multiplex (Panel B) assays. Testing with singleplex assay, we found that OPV2 in fecal samples of household contacts was associated with shedding poliovirus overall and among non-vaccinated individuals. In contrast, among vaccinated individuals, we observed that presence of any of the three serotypes in fecal samples of household contacts was associated with OPV shedding.

**Table 2:** Association between OPV serotypes in fecal samples with OPV shedding of vaccinated and non-vaccinated household contacts using singleplex and multiplex assays.

Characteristic	Total		Fecal sample with OPV		Fecal sample without OPV		p value^a^
	n/total	(%)	n/total	(%)	n/total	(%)	
Presence of OPV in at least one sample from a household contact during the same month of sample collection.							
**Panel A: Singleplex assay**							
**1. Overall**							
Poliovirus 1	57/2500	(2.3)	4/142	(2.8)	53/2358	(2.2)	0.659
Poliovirus 2	177/2500	(7.1)	43/142	(30.3)	134/2358	(5.7)	<0.001
Poliovirus 3	92/2500	(3.7)	8/142	(5.6)	84/2358	(3.6)	0.203
**2. tOPV vaccinated**							
Poliovirus 1	6/467	(1.3)	3/75	(4.0)	3/392	(0.8)	0.023
Poliovirus 2	30/467	(6.4)	16/75	(21.3)	14/392	(3.6)	<0.001
Poliovirus 3	5/467	(1.1)	5/75	(6.7)	0/392	(0.0)	<0.001
**3. Not vaccinated**							
Poliovirus 1	51/2033	(2.5)	1/67	(1.5)	50/1966	(2.5)	0.589
Poliovirus 2	147/2033	(7.2)	27/67	(40.3)	120/1966	(6.1)	<0.001
Poliovirus 3	87/2033	(4.3)	3/67	(4.5)	84/1966	(4.3)	0.935
Poliovirus 1	57/2500	(2.3)	4/142	(2.8)	53/2358	(2.2)	0.659
**Panel B: Multiplex assay**							
**1. Overall**							
Poliovirus 1	16/256	(6.3)	5/76	(6.6)	11/180	(6.1)	0.888
Poliovirus 2	20/256	(7.8)	10/76	(13.2)	10/180	(5.6)	0.038
Poliovirus 3	16/256	(6.3)	9/76	(11.8)	7/180	(3.9)	0.016
**2. tOPV vaccinated**							
Poliovirus 1	4/75	(5.3)	4/48	(8.3)	0/27	(0.0)	0.123
Poliovirus 2	8/75	(10.7)	7/48	(14.6)	1/27	(3.7)	0.143
Poliovirus 3	6/75	(8.0)	6/48	(12.5)	0/27	(0.0)	0.055
**3. Not vaccinated**							
Poliovirus 1	12/181	(6.6)	1/28	(3.6)	11/153	(7.2)	0.479
Poliovirus 2	12/181	(6.6)	3/28	(10.7)	9/153	(5.9)	0.345
Poliovirus 3	10/181	(5.1)	3/28	(10.7)	7/153	(4.6)	0.191

^a^ χ2 test

When we analyzed data of samples processed by multiplex assay (Panel B), we found association between OPV2 and OPV3 in fecal samples of household contacts with shedding.

Association of number of OPV doses and OPV in fecal samples of children 36 months and younger is shown in S1 Table using singleplex assay. Although the likelihood of OPV shedding decreased with increasing number of OPV doses, the decrease was not statistically significant.

Association of individual characteristics with OPV in fecal samples using Cox proportional hazards regression for recurrent events models accounting for individual clustering overall (Model 1), for tOPV vaccinated (Model 2) and non-vaccinated (Model 3) individuals are shown in Table 3 when samples were tested with singleplex assay. In the first model we analyzed samples from all study participants. Variables associated with a higher likelihood of shedding any poliovirus were having received tOPV in the previous NHW, having shed Sabin poliovirus 3 during the previous month and having at least one household member who shed Sabin poliovirus 2 during the same month. Among vaccinated individuals, variables associated with a higher likelihood of shedding any poliovirus were having shed Sabin poliovirus 3 during the previous month and having at least one household member who shed poliovirus Sabin type 1, 2 or 3 during the same month. Among non-vaccinated individuals, variables associated with a higher likelihood of shedding any poliovirus were having shed Sabin poliovirus 1 or Sabin poliovirus 3 during the previous month and having at least one household member who shed Sabin poliovirus 2 during the same month.

**Table 3:** Association of individual characteristics with OPV in fecal samples using Cox proportional hazards regression for recurrent events models accounting for individual clustering testing with singleplex assay.

Parameter	Model^a^ 1(Overall shedding)	Model^a^ 2(Vaccinated individuals)	Model^a^ 3(Non-vaccinated individuals)
	aHR^b^	aHR^b^	aHR^b^
	(95%CI)^c^	(95%CI)^c^	(95%CI)^c^
No. of samples included in each model	2279	415	1864
OPV^d^ vaccination during the previous NHW	2.8***	-----	-----
	(1.7–4.6)	-----	-----
Male	1.1	1.0	1.0
	(0.8–1.5)	(0.6–1.7)	(0.6–1.7)
Age (years)	1.0	1.1	1.0
	(1.0–1.0)	(0.8–1.3)	(1.0–1.0)
IPV^e^ vaccination	1.0	0.9	1.1
	(0.9–1.2)	(0.7–1.2)	(0.9–1.3)
*Shedding during the previous month to sample collectio*			
PV^f^1	1.5	1.3	2.4*
	(0.7–3.4)	(0.4–4.4)	(1.1–5.0)
PV^f^2	1.1	0.6	2.1
	(0.5–2.1)	(0.2–1.5)	(0.9–5.3)
PV^f^3	3.4***	2.3*	3.8**
	(2.0–5.9)	(1.0–5.2)	(1.7–8.5)
*At least one shedding household contact during the same month of sample collection*			
PV^f^1	0.9	8.1**	0.4
	(0.3–2.9)	(2.0–32.7)	(0.1–2.6)
PV^f^2	7.9***	3.9***	12.7***
	(5.1–12.2)	(2.1–7.0)	(7.0–23.1)
PV^f^3	1.4	8.1***	0.8
	(0.6–3.5)	(3.1–21.6)	(0.2–2.4)
At least one contact received OPV less than 30 days previous to sample collection	0.9	0.8	0.8
	(0.6–1.4)	(0.3–1.8)	(0.4–1.3)

^a^ Cox proportional hazards model for recurrent events clustered by participant.

^b^ aHR = Adjusted hazard ratio.

^c^95%CI = 95% confidence intervals.

^d^ OPV = Oral polio vaccine.

^e^ IPV = Inactivated polio vaccine.

^f^ PV = Poliovirus.

*p<0.05,

**p<0.01 and

***p<0.001.

Association of individual characteristics with OPV in fecal samples overall (Model 1), for tOPV vaccinated (Model 2) and non-vaccinated (Model 3) among children 36 months old and younger testing with singleplex assay is shown in S2 Table. In the first model we analyzed samples from all children 36 months old and younger. Variables associated with a higher likelihood of shedding were having shed Sabin poliovirus 3 during the previous month and having at least one household member who shed Sabin poliovirus 1, 2 or 3 during the same month. We did not find association between number of OPV doses received during the lifetime of the child and the likelihood of OPV shedding. Among vaccinated individuals, variables associated with a higher likelihood of shedding any poliovirus were age and having at least one household member who shed poliovirus Sabin type 1, 2 or 3 during the same month. Among non-vaccinated individuals, variables associated with a higher likelihood of shedding any poliovirus were age, having shed Sabin poliovirus 3 during the previous month and having at least one household member who shed Sabin poliovirus 2 during the same month. There were one and two samples from household contacts with Sabin type 1 and 3 respectively, none of which was associated with OPV shedding.

Association of individual characteristics with OPV in fecal samples using Cox proportional hazards regression for recurrent events models accounting for individual clustering overall (Model 1), for tOPV vaccinated (Model 2) and non-vaccinated (Model 3) for the 256 individuals whose samples were processed by serotype-specific multiplex rRT-PCR is shown in Table 4. In the first model we analyzed samples from all 256 study participants. Variables associated with a higher likelihood of shedding any poliovirus were age, having shed Sabin poliovirus 1 or 2 during the previous month and having at least one household member who shed Sabin poliovirus 2 during the same month. Among vaccinated individuals, variables associated with a higher likelihood of shedding any poliovirus were male sex and having shed Sabin poliovirus 1 during the previous month. Among non-vaccinated individuals, variables associated to a higher likelihood of shedding any poliovirus were having shed Sabin poliovirus 1 or Sabin poliovirus 2 during the previous month and having at least one household member who shed poliovirus Sabin type 1, 2 or 3 during the same month.

**Table 4:** Association of individual characteristics with OPV in fecal samples by serotype-specific multiplex rRT-PCR using Cox proportional hazards regression for recurrent events models accounting for individual clustering among 256 samples.

Parameter	Model^a^ 1 (Overall shedding)	Model^a^ 2 (Vaccinated individuals)	Model^a^ 3 (Non-vaccinated individuals)
	aHR^b^	aHR^b^	aHR^b^
	(95%CI)^c^	(95%CI)^c^	(95%CI)^c^
No. of samples included in each model	244	66	178
OPV^d^ vaccination	1.274	-----	-----
	(0.77–2.12)	-----	-----
Male	0.540	0.412*	0.540
	(0.27–1.09)	(0.20–0.86)	(0.26–1.12)
Age (years)	0.947*	1.482	0.938
	(0.90–0.99)	(0.79–2.77)	(0.87–1.01)
IPV^e^ vaccination	1.070	0.734	1.048
	(0.86–1.34)	(0.52–1.03)	(0.73–1.51)
*Shedding during the previous month to sample collection*			
PV^f^1	3.419***	3.176***	5.563*
	(1.70–6.88)	(1.62–6.23)	(1.35–22.85)
PV^f^2	2.845**	2.208	3.339*
	(1.34–6.02)	(0.92–5.28)	(1.31–8.49)
PV^f^3	-----^g^	-----^h^	-----^i^
		
*At least one shedding household contact during the same month of sample collection*			
PV^f^1	0.370	0.486	0.036***
	(0.12–1.19)	(0.16–1.46)	(0.01–0.20)
PV^f^2	4.642***	3.845	11.04***
	(1.34–16.13)	(0.47–31.30)	(3.19–38.21)
PV^f^3	1.300	0.898	7.659**
	(0.46–3.66)	(0.15–5.49)	(1.99–29.47)
At least one contact received OPV less than 30 days previous to sample collection	0.708	0.623	0.789
	(0.40–1.26)	(0.25–1.57)	(0.31–2.03)

^a^ Cox proportional hazards model for recurrent events clustered by participant.

^b^ aHR = Adjusted hazard ratio.

^c^95%CI = 95% confidence intervals.

^d^ OPV = Oral polio vaccine.

^e^ IPV = Inactivated polio vaccine.

^f^ PV = Poliovirus,

^g^ There were 49 instances with OPV3 in the sample collected during the previous month, none of which was associated to OPV shedding.

^h^ There were 35 instances with OPV3 in the sample collected during the previous month, none of which was associated to OPV shedding.

^i^ There were 14 instances with OPV3 in the sample collected during the previous month, none of which was associated to OPV shedding.

*p<0.05,

**p<0.01 and

***p<0.001.

## Discussion

We describe the individual characteristics associated with fecal poliovirus shedding after receipt of OPV among 72 young children and their 144 household contacts over a 12-month period, in an environment of mixed vaccination against polio (IPV + OPV). We found that individual variables associated with poliovirus shedding differed between participants who had received tOPV during the previous NHW week and those who had not. We tested for OPV in stool samples using a singleplex rRT-PCR assay and with a two-step, OPV serotype-specific multiplex rRT-PCR in order to increase specificity of the test. Both assays showed that Sabin poliovirus 2 in stool samples of household contacts was associated with poliovirus shedding among non-vaccinated individuals. We did not observe association of OPV in fecal samples with number of OPV doses received during lifetime.

In a previous manuscript in which we analyzed community circulation rather than individual shedding from this same study we observed that Sabin poliovirus was detected up to 6 months after a NHW among enrolled children younger than 3 years old, and up to 7 months in their household contacts that included both children and adults. Serotype 2 circulated longer and at higher rates in the community after a NHW than serotypes 1 and 3 [6]. In this study, we confirmed these results using Kaplan Meier survival analyses to describe the positive sample probability of samples from vaccinated and non-vaccinated participants. Importantly, however, when we analyzed the 256 samples tested with multiplex assay we did not confirm our previous finding of extended OPV2 shedding. As has been previously described, sequencing showed extended shedding was due to enterovirus C (NPEV-C) strain [12]. Furthermore, samples that contained NPEV-C were collected during summer and fall months when circulation rates of NPEV-C are higher. The study of Holubar et. al. [12] and the present analysis using Kaplan Meier curves on samples tested with the multiplex assay raise doubts on the previous finding of a longer period of time between OPV vaccination campaigns and identification of OPV2 in fecal samples.

Assessment of individual risk of shedding supports results of our previous study in which we analyzed community shedding and found that OPV2 was more likely to transmit within the household. In the previous study we presented results from positive samples and used a different definition of shedding, as we were studying community circulation of OPV [6]. When we analyzed only positive samples in our previous manuscript [7] we found that all 3 Sabin poliovirus serotypes were shed early by vaccinated children in roughly equivalent proportions. In contrast more than 85% of the community acquired Sabin poliovirus among both children and adults were serotype 2. Using a different approach in the present study and with fecal samples as study unit, we analyzed by Cox proportional hazards regression the individual characteristics associated with shedding overall and among tOPV vaccinated and non-vaccinated children and subjects older than 5 years of age. We found that OPV in fecal samples tested by either singleplex or multiplex assays was associated with Sabin poliovirus 2 shed by persons living in the same household and whose sample was collected in the same month. We observed similar results when we analyzed the subgroup of children 36 months old and younger.

Available data support the notion that serotype 2 is “fitter” than the other two serotypes. Initial vaccination studies with OPV showed that serotype 2 had higher shedding rates and caused immunological interference with the other two serotypes. As a consequence, the amount of virus type 2 was reduced in tOPV [2]. Sabin poliovirus 2 is largely shed by vaccinated individuals and remains in the community through transmission to other members [2, 14, 15]. In studies done during the second half of last century in the US and Russia, it was found that serotype 2 had a higher probability of being shed by household contacts of vaccinated individuals with the existing vaccination formulas [2, 15]. Furthermore, cVDPV2 has been associated with paralytic disease in recent years. The two most important outbreaks are those that affected Nigeria and Pakistan for several years, where there was sustained circulation of the virus consecutively during 10 and 4 years, respectively [1].

We did not find association between shedding of poliovirus and breastfeeding of participants, number of doses of IPV that vaccinated individuals received, household conditions (sanitary services, type of floor, crowding), as has been described in the literature [16]. This is probably due to the population being very homogenous and with high vaccination coverage [17].We observed association with age only when we analyzed the subgroup of children 36 months old and younger. Older age was associated with OPV in fecal samples in the subgroup of older vaccinated children. This is probably related to the fact that children should have received two IPV doses in order to be vaccinated during the NHW. Therefore, older children were more likely to be vaccinated (in the NHW previous to sample collection) and therefore have OPV in their fecal samples. In contrast, younger age was associated with OPV in fecal samples among the subgroup of non-vaccinated (in the NHW previous to sample collection) children, probably due to higher likelihood of transmission to children with fewer lifetime doses of OPV and therefore less intestinal immunity.

In a previous study [18], we have described that the presence of mutant variants and variants per sample was quite low in a subset of the samples we collected; we therefore consider that it is unlikely that there were any VDPVs among our samples.

Our study has several limitations. History of vaccination with OPV might have not been totally accurate since this information is not registered in the child’s Health Card and we relied on the information provided by the caregiver (in most cases the mother). However, this information has been found to be closely associated with serological findings. [6]. In addition, the time elapsed since the last NHW was short and OPV is the only vaccine administered orally. Secondly, the PCR that we used is more sensitive for serotype 2 than for 1 and 3 [12, 19]. In addition, the singleplex PCR for serotype 2 used for the main analysis was later found to have some cross-reactivity with nonpolio enterovirus type C [12]. However, as described, we repeated the analysis with 256 samples that were analyzed with a new two-step, serotype-specific multiplex rRT-PCR that does not have any known cross-reactivity with NPEV-C, with results similarly showing that OPV in samples from all participants is associated with shedding of OPV2 by household contacts. From the methodological point of view, Cox proportional hazards regression for recurrent events model accounting for individual clustering requires that the supposition of proportional risks be fulfilled. In order to comply with this, the time period to each event for the same individual was analyzed separately and adjusted for the fact that time periods for each individual were independent. We dealt with the existence of statistical dependence between the time to each event in the same individual using a model of shared fragility with gamma distribution.

Our results provide important evidence regarding the circulation of poliovirus in a mixed vaccination context (IPV+OPV) which mimics the “transitional phase” that occurs when countries use both vaccines simultaneously. Shedding of OPV2 by household contacts is more likely the source of infection of non-vaccinated contacts.

## Supporting information

S1 Dataset Data set of patient characteristics and results of singleplex assays. (XLSX)

S2 Dataset Data set of patient characteristics and results of multiplex assays. (XLSX)

S3 Dataset Description of study variables contained in data sets S3 and S4. (XLSX)

S1 Table Presence of OPV in fecal samples by number of OPV vaccine doses among children 36 months old and younger. (DOCX)

S2 Table Association of individual characteristics with OPV in fecal samples using Cox proportional hazards regression for recurrent events models accounting for individual clustering among children 36 months old and younger. (DOCX)
