# Supplementary material for: Assessing the individual risk of fecal poliovirus shedding among vaccinated and non-vaccinated subjects following national health weeks in Mexico
Source: PLoS One. 2017 Oct 12;12(10):e0185594. doi: 10.1371/journal.pone.0185594 (PMC5638237; doi:10.1371/journal.pone.0185594)
Supplement: S1 Table — (DOCX) [file pone.0185594.s004.docx]

S1 table. Presence of OPV in fecal samples according to number of OPV vaccine doses among children 36 months old and younger

| Number of OPV vaccine doses | Total | OPV in fecal sample  n (%) | No OPV in fecal sample  n (%) | p value |
| --- | --- | --- | --- | --- |
| Non vaccinated | 344 | 37 (10.76) | 307 (89.24) | 0.536 |
| 1 dose | 274 | 37 (13.50) | 237 (86.50) |  |
| 2 doses | 152 | 19 (12.50) | 133 (87.50) |  |
| 3 doses | 64 | 5 (7.81) | 59 (92.19) |  |
| Total | 834 | 98 | 736 |  |
