# Supplementary material for: Assessing the individual risk of fecal poliovirus shedding among vaccinated and non-vaccinated subjects following national health weeks in Mexico
Source: PLoS One. 2017 Oct 12;12(10):e0185594. doi: 10.1371/journal.pone.0185594 (PMC5638237; doi:10.1371/journal.pone.0185594)
Supplement: S2 Table — (DOCX) [file pone.0185594.s005.docx]

S2 Table. Association of individual characteristics with OPV in fecal samples using Cox proportional hazards regression for recurrent events models accounting for individual clustering among children 36 months old and younger.

|  | **Model^a^ 1**  **(Overall shedding)** | **Model^a^ 2**  **(Vaccinated individuals)** | **Model^a^ 3**  **(Non-vaccinated individuals)** |
| --- | --- | --- | --- |
|  | **aHR^b^** | **aHR^b^** | **aHR^b^** |
|  | **(95%CI)^c^** | **(95%CI)^c^** | **(95%CI)^c^** |
| No. of samples included in each model | 756 | 366 | 390 |
| Non vaccinated | 1 | 1 | 1 |
|  | Reference | Reference | Reference |
| 1 dose OPV**^d^** | 0.959 | 0.524 | 2.635 |
|  | (0.55-1.67) | (0.27-1.01) | (0.76-9.10) |
| 2 doses OPV | 0.886 | 0.55 | 2.48 |
|  | (0.48-1.64) | (0.26-1.14) | (0.55-11.28) |
| 3 doses OPV | 0.549 | 0.258 | 2.357 |
|  | (0.24-1.24) | (0.07-1.01) | (0.17-32.93) |
| Male | 1.04 | 0.84 | 1.179 |
|  | (0.68-1.58) | (0.47-1.51) | (0.49-2.83) |
| Age (months) | 1.015 | 1.076*** | 0.901* |
|  | (0.98-1.05) | (1.04-1.11) | (0.81-1.00) |
| IPV^e^ vaccination | 1.185 | 0.767 | 1.818 |
|  | (0.88-1.59) | (0.55-1.08) | (0.94-3.54) |
| *Shedding during the previous month to sample collection* | | | |
| PV^f^1 | 1.618 | 1.02 | 1.721 |
|  | (0.73-3.60) | (0.27-3.88) | (0.65-4.56) |
| PV^f^2 | 1.27 | 0.656 | 2.794 |
|  | (0.63-2.57) | (0.24-1.78) | (0.79-9.86) |
| PV^f^3 | 2.357** | 1.519 | 3.823** |
|  | (1.39-4.00) | (0.48-4.80) | (1.66-8.79) |
| *At least one shedding household contact during the same month of sample collection* | | | |
| PV^f^1 | 21.91** | 20.81* | ------^g^ |
|  | (2.99-160.43) | (1.94-223.41) |  |
| PV^f^2 | 3.389*** | 2.353** | 7.179** |
|  | (1.89-6.08) | (1.27-4.35) | (1.80-28.62) |
| PV^f^3 | 9.002*** | 11.82*** | ------ ^h^ |
|  | (3.05-26.59) | (3.54-39.50) |  |
| At least one contact received OPV less than 30 days previous to sample collection | | | |
| PV2 | 0.671 | 0.639 | 0.355 |
|  | (0.22-2.09) | (0.20-2.03) | (0.07-1.73) |

^a^ Cox proportional hazards model for recurrent events clustered by participant. ^b^ aHR= Adjusted hazard ratio. ^c^95%CI= 95% confidence intervals. ^d^ OPV=Oral polio vaccine. ^e^ IPV=Inactivated polio vaccine. ^f^ PV= Poliovirus, ^g^ One sample from a household contact with OPV1 was not associated to OPV shedding. ^h^ There were 2 samples from household contacts with OPV3, none of which were associated to OPV shedding.*p<0.05, **p<0.01 and ***p<0.001.

.
